# The significance of uterine artery embolization in the treatment of utero‐cervical cancer: A single case report

**DOI:** 10.1002/ccr3.9458

**Published:** 2024-09-22

**Authors:** Preeti Kumari Yadav, Abhimanyu Sharma, Muhammad Ali, Nida Khan, Jubran Al Balushi, Hajrah Farooq, Sree Abhilekha Purohit, Sofia Ali, Malavika Jayan, Archit Kumar Nigam, Mansi Singh

**Affiliations:** ^1^ Department of Medicine Cama and Albless Hospital Mumbai India; ^2^ Department of Medicine SGRD Institute Of Medical Sciences And Research Amritsar India; ^3^ Department of Medicine Islamic International medical college, Riphah University Rawalpindi Pakistan; ^4^ Department of Medicine Jinnah Sindh Medical University Pakistan; ^5^ Department of Medicine University College Dublin Dublin Ireland; ^6^ Department of Medicine Shadan Institute of Medical Sciences Hyderabad India; ^7^ Department of Medicine, Peninsula Medical School University of Plymouth Plymouth UK; ^8^ Bangalore Medical College and Research Institute Bangalore Karnataka India; ^9^ Department of Medicine and Radiology Regency Health Kanpur India; ^10^ Department of Medicine Bogomolets National Medical University Kyiv Ukraine

**Keywords:** human papillomavirus (HPV), interventional radiology, uterine artery embolisation (UAE), uterocervical cancer, vaginal hemorrhage

## Abstract

**Key Clinical Message:**

Uterine artery embolization demonstrated significant efficacy in the treatment of utero‐cervical cancer. This minimally invasive procedure holds promise as a valuable adjunct therapy, potentially offering improved outcomes and reduced morbidity in select cases. Further research is warranted to validate its broader clinical utility.

**Abstract:**

Vaginal bleeding is a common complication of Cervical cancer that can be considered a critical emergency. Conventional hemostatic treatments may occasionally help reduce the bleeding but are not an effective long‐term solution. Uterine Artery Embolization, a minimally invasive intervention, can halt the bleeding, achieving hemostasis, while removing many of the complications that alternative interventions carry. We outline a case study of a patient with extensive vaginal bleeding who had uterocervical malignancy and talk about the benefits of uterine artery embolisation for therapeutic management.

## 1 INTRODUCTION

Cervical cancer was ranked fourth most common cancer among women and continues to pose a significant burden to global health. World Health Organization (WHO) recorded 604,000 new cases in 2020, in addition to 342,000 deaths, 90% being women from low and middle‐income countries. This is likely due to the disparity in access to health services, such as Human Papillomavirus (HPV) vaccination and cervical screening, exacerbating mortality rates.^1^ It is clearly established that persistent infection with HPV is the main cause of cervical cancer, with 98.7% of cases being HPV‐positive cases.^2^

Progression from HPV infection to cervical cancer takes roughly 15–20 years.^1^ Furthermore, the disease is asymptomatic in the early stages, adding to the difficulty of detection and the necessity of screening. Therefore, the most important interventions adopted for Cervical Cancer are primary and secondary prevention, which prove to be the most effective ways to lessen the incidence of cervical cancer and related mortality^3^; The benefits of the Pap smear are well established and show that screened patients have a 38% lower risk of death than non‐screened patients.^4^ Additionally, studies indicate that the introduction of HPV vaccinations in 2006 was the fundamental cause of the 29% fall in average annual cervical cancer incidence rate from 2004–2006 to 2011–2014.^5^

Despite these protective measures, cervical cancer remains a burden on global health, particularly in regions where access to preventative healthcare is limited. For this reason, it is vital to explore the interventions for cervical cancer and the many complications it can present, one of the most prominent being vaginal bleeding. This is due to the invasive nature of the cancer cells that continuously proliferate deeper into the tissue compromising the integrity of blood vessels as well as forming a mass that can erode through the surface epithelium leading to ulcerations and bleeding.^6^

In 1995, interventional radiologists developed Uterine Artery Embolisation (UAE), a minimally invasive technique that was first used to treat uterine fibroids and post‐partum hemorrhages. It functions to selectively occlude the uterine artery with the aim of reducing blood flow to targeted areas, effectively shrinking the tumor and minimizing bleeding.^7^ It has since been well integrated and accepted into normal medical practice more so in the reduction of uterine fibroids as well as controlling bleeding as it provides an alternative option to invasive surgeries such as hysterectomy and myomectomy. Additionally, in the case of cervical cancer, UAE is mainly adopted for the management of pelvic bleeding and palliative care where more invasive surgeries are not feasible.^8^ Here, we describe a case where Uterine Artery Embolization was used to successfully treat cervical cancer and, more specifically, cervical hemorrhage.

## 2 CASE HISTORY

A 35‐year‐old patient presented to our center with a mass per vagina, accompanied by severe vaginal bleeding and pelvic discomfort. The onset of abnormal vaginal bleeding occurred 2 months prior to admission, with no personal or family history of cervical cancer.

Upon current clinical examination, the patient exhibited profuse cervical bleeding without clotting. Vital signs revealed a blood pressure of 90/50 mmHg and a heart rate of 100 beats per minute.

## 3 METHODS

**FIGURE 1:**
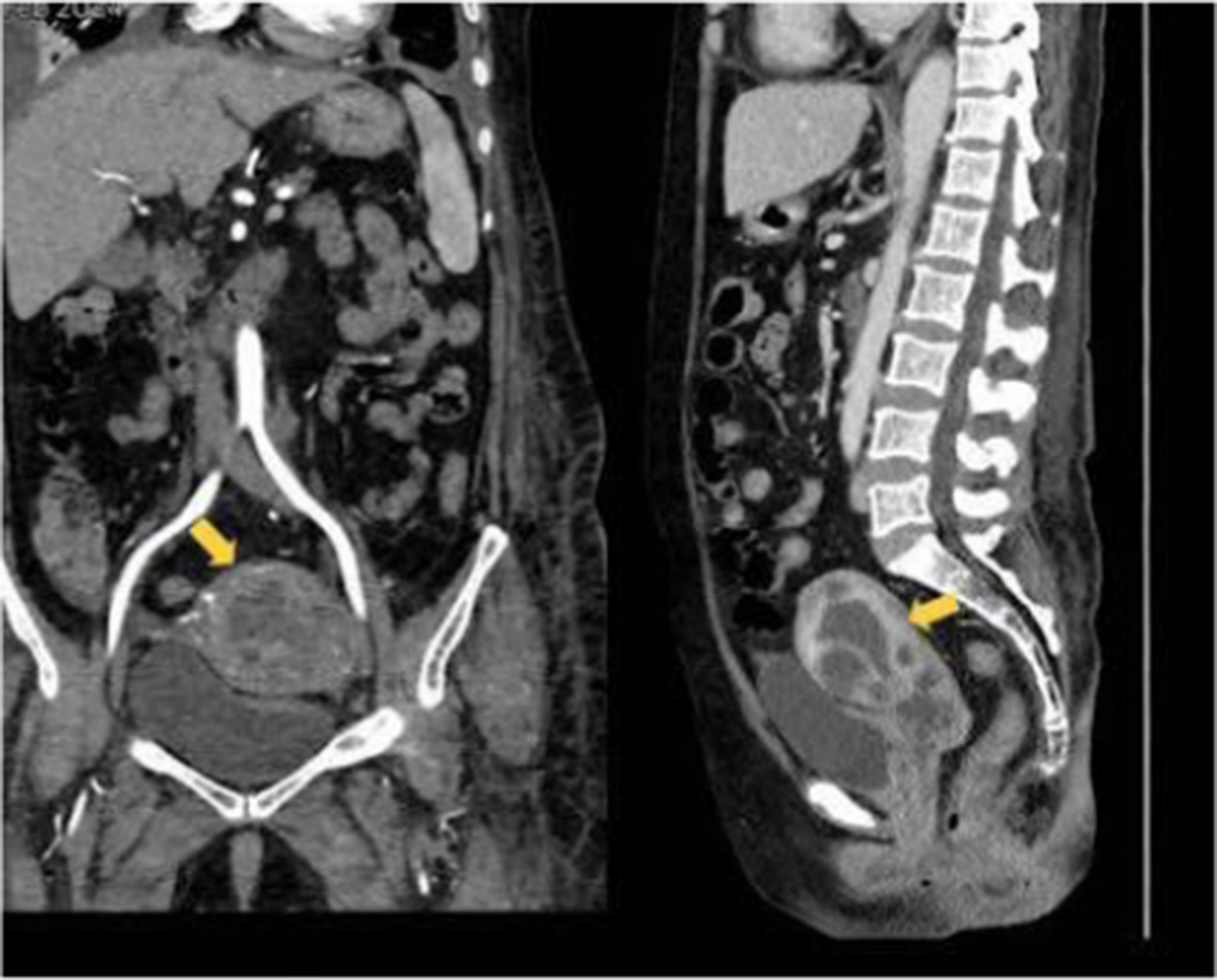
Coronal and Sagittal CT scans of the lower abdominal region. The yellow arrows indicates the 32 × 45 × 27 mm intramural mass on the posterior cervix wall, with infiltrative extensions into the lateral and anterior walls.

Routine laboratory investigations revealed severe secondary anemia with a hemoglobin level of 7 mg/dL. Transvaginal ultrasound and CT findings suggested the presence of a 32 × 45 × 27 mm intramural mass on the posterior cervix wall (as shown in Figure 1), with infiltrative extensions into the lateral and anterior walls, along with two images of external iliac lymph nodes (13 mm on the right side and 14 mm on the left side).

## 4 CONCLUSION AND OUTCOMES

**FIGURE 2:**
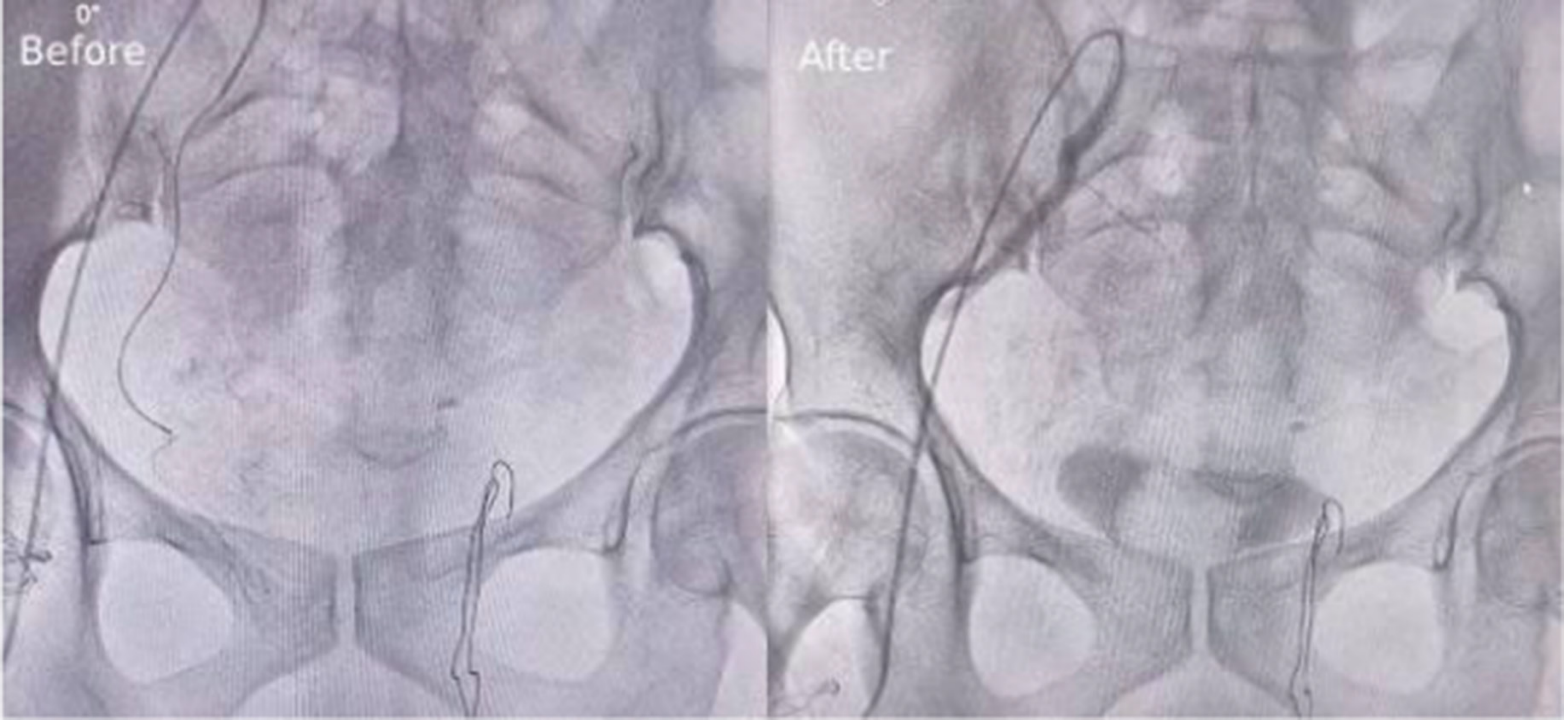
Right Uterine Artery, pelvic arteriography: Before and after embolization.

**FIGURE 3:**
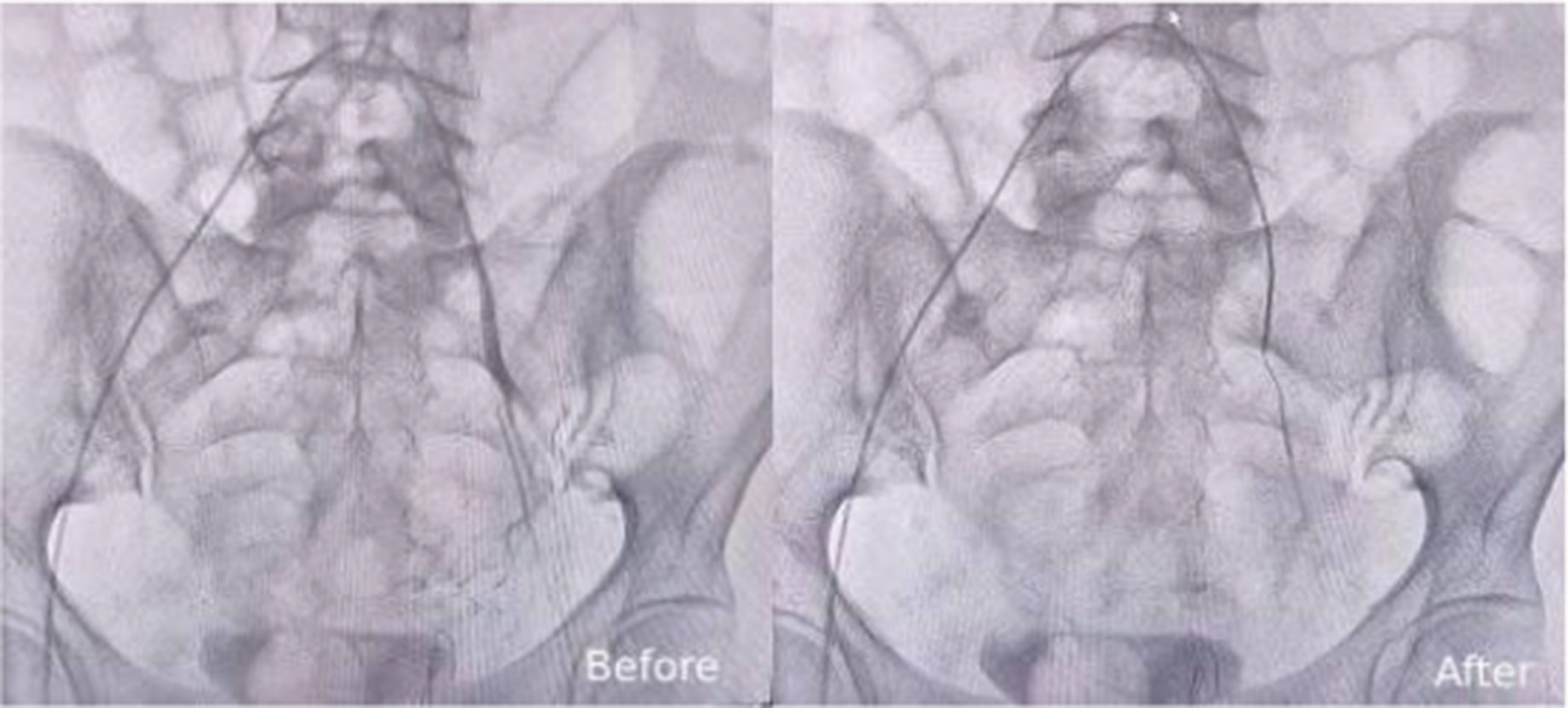
Left Uterine Artery, pelvic arteriography: Before and after embolization.

Based on these findings, a suspected diagnosis of IIB stage cervical cancer was made. A cervix biopsy was performed under local anesthesia, followed by initiation of hemostatic treatment comprising local and intravenous Adrenostasine, Fitomenadione, and Ethamsylate. Blood transfusion of 2 units was initiated to correct the anemia. But despite 2 days of hemostatic therapy, the vaginal bleeding persisted, prompting uterine artery embolization until blood flow stasis was achieved. Uterine artery embolization, a minimally invasive interventional radiology technique, was performed using a unilateral brachial approach. This approach allowed immediate patient mobilization postintervention and required only one puncture. After local anesthesia with Xylocaine, Embolospheres combined with a contrast substance were injected, selectively catheterizing each vessel supplying the tumor. Vaginal bleeding ceased within 24 hours post‐intervention. Figures 2 and 3 show pelvic arteriography findings in the right and left uterine arteries before and after embolization.

The conservative treatment provided effective relief of the patient's symptoms, aided by the robust collateral blood supply characteristic of the pelvic region. Pelvic pain was managed with anti‐inflammatory and antibiotic medications. External radiotherapy totaling 26.2 GY, combined with 6 weeks post‐radiotherapy, curietherapy, was subsequently administered. A complete lymphadenocolpohysterectomy (Wertheim technique) was performed, with histopathological analysis confirming the presence of squamous cell cervical cancer.

Cervical hemorrage poses a significant risk to both morbidity and mortality across various gynacological disorders. Proving to be a substantial obstacle for gynacologists. Highlighting the minimally invasive nature of Uterine Artery Embolization and its role in improving outcomes for these patients by providing an alternative with a reduced risk of surgical morbidity.

## 5 DISCUSSION

There are various techniques used in cases of cervical hemorrhage, most of which are considered emergency procedures because of the severity of the complications that arise, such as anemia and hemorrhagic shock. Management options like vaginal packing, kaolin‐based hemostatic gauze and off‐label Tranemix acid are useful to stop the bleeding in an emergency. However, these do not provide a definitive solution and are sometimes insufficient measures for more severe cases.^9^ There are alternative, more invasive, treatment options; however, they pose many intraoperative and postoperative challenges. Radiation therapy may be considered, but the complications and risks associated with this procedure make it unfavorable.^10^ Resections pose many risks as well; for example, Trachelectomy is associated with deep dyspareunia and recurrent candidiasis due to the cervical cerclage suture acting as a niche for vaginal organisms, while, Hysterectomy is associated with lymphoedema and decreased sexual drive.^11^ Furthermore, when evaluating the psychological effects of the surgical wound, infertility and anesthetic risk, other minimally invasive alternatives become more desirable. There are reports of introducing endoscopic hemostatic forceps for cervical hemorrhage management that prove it to be a suitable minimally invasive procedure, however, its conventional use is in the abdomen and its gynecological use requires further research.^12^

Hypogastric artery ligation has historically been the standard method for managing severe hemorrhage in cervical cancer, with success rates reported in series varying greatly between 40% and 100%. However, this approach led to severely deformed pelvic anatomy as a result of cancer recurrence and radiotherapy, posing a significant concern to doctors. Furthermore, due to the invasive nature of this procedure, there are increased operative difficulties and anesthetic risks in critically ill paitents.^7^ This prompted the introduction of transcatheter arterial embolization in the mid‐1970s, which revolutionized the management of intractable pelvic hemorrhage in patients with gynecologic malignancies. Although embolization of the anterior branch of the internal iliac artery has gained quick popularity because of its low rates of death and complications, it has been linked to unfavorable outcomes like bladder ischemia, vesicovaginal fistula, and neurological impairments. However, super‐selective embolization of the uterine artery led to the reduction of these complications.^13^, ^14^, ^15^, ^16^

Several studies have highlighted the advantages of UAE in controlling bleeding in pelvic neoplasms. Pisco et al. a study of 108 patients with intractable cervical hemorrhage demonstrated that embolization of the internal iliac arteries led to complete hemorrhage control in 69% of patients and restricted control in 21%.^17^ Yamashita et al. achieved temporary cessation of bleeding in 100% of patients suffering from malignant tumors, while Mihmanli et al. demonstrated effective control of vaginal bleeding in individuals with gynecological cancers through the use of polyvinyl alcohol particles in arterial embolization.^16^, ^18^ Serdar et al. reported temporary bleeding control in 100% of patients with terminal‐stage cervical cancer, with two developing vesicovaginal fistulae, the cause of which remained unclear.^19^ These limited studies suggest that arterial embolization is key in managing pelvic bleeding and presents a viable option over more invasive methods.

Arterial embolization offers several advantages, including rapid hemorrhage control, which allows for quicker stabilization of a patient's condition, especially if anemic. It is a minimally invasive procedure with a small unilateral brachial incision, the lack of a significant‐sized wound and effective reduction of bleeding allows for a short hospital stay, quick recovery and reintegration back into society. Furthermore, UEA removes the anesthetic and prolonged operation risks, which would increase patients' morbidity, especially those in advanced stages of the disease.^8^ The rest of the uterus is not affected as there is sufficient collateral blood supply to sustain it and therefore fertility is not affected.^7^ Moreover, UAE is associated with lower rebleeding incidence compared to surgical ligation, improved patient condition, decreased tumor size due to diminished tumor feeding, and reduced intraoperative bleeding during subsequent surgical interventions. Immediate reduction in blood loss eliminates the need for blood transfusion and prevents potential transfusion‐related complications.^18^, ^19^

Noteworthy complications that may arise include pain due to tissue necrosis, however, this can be managed with 1–2 days of analgesics or by superior hypogastric nerve block before the procedure.^20^ Similar to all procedures there is also a small risk of infection, Rajan et al. reported out of 410 patients who received UAE 5 suffered from an intrauterine infection requiring IV antibiotics and/or surgery.^21^ In conclusion, uterine arterial embolization provides a reliable and successful method for managing significant bleeding in patients with cervical cancer.

## AUTHOR CONTRIBUTIONS

**Preeti Kumari Yadav:** Conceptualization; investigation; software; writing – original draft. **Abhimanyu Sharma:** Conceptualization; funding acquisition; supervision; validation. **Muhammad Ali:** Funding acquisition; software; supervision; validation. **Nida Khan:** Conceptualization; funding acquisition; supervision; visualization. **Jubran Al Balushi:** Conceptualization; data curation; investigation; supervision. **Hajrah Farooq:** Conceptualization; formal analysis; funding acquisition; resources. **Sree Abhilekha Purohit:** Conceptualization; data curation; formal analysis; funding acquisition; methodology. **Sofia Ali:** Funding acquisition; investigation; methodology; visualization. **Malavika Jayan:** Conceptualization; formal analysis; investigation; validation; writing – review and editing. **Archit Kumar Nigam:** Resources; software; supervision; validation. **Mansi Singh:** Conceptualization; data curation; investigation; project administration; supervision; writing – original draft.

## FUNDING INFORMATION

None.

## CONFLICT OF INTEREST STATEMENT

The authors declare no conflicts of interest.

## CONSENT

Written informed consent was obtained from the patient to publish this report in accordance with the journal's patient consent policy.

## Data Availability

The datasets analyzed during the current study are available from the corresponding author upon reasonable request. Additionally, comprehensive literature sources used for the literature review are cited appropriately within the manuscript.

## References

[1] World Health Organization . Cervical cancer. Accessed March 1, 2024. https://www.who.int/news‐room/fact‐sheets/detail/cervical‐cancer

[2] Wang X , Huang X , Zhang Y . Involvement of human papillomaviruses in cervical cancer. Front Microbiol. 2018;9:2896. doi:10.3389/fmicb.2018.02896 30546351 PMC6279876

[3] Pimple SA , Mishra GA . Global strategies for cervical cancer prevention and screening. Minerva Ginecol. 2019;71(4):313‐320. doi:10.23736/S0026-4784.19.04397-1 30808155

[4] Luu XQ , Lee K , Jun JK , et al. Effect of pap smears on the long‐term survival of cervical cancer patients: a nationwide population‐based cohort study in Korea. Epidemiol Health. 2022;44:e2022072. doi:10.4178/epih.e2022072 36108672 PMC9943631

[5] Guo F , Cofie LE , Berenson AB . Cervical cancer incidence in young U.S. females after human papillomavirus vaccine introduction. Am J Prev Med. 2018;55:197‐204. Accessed March 1, 2024. https://www.sciencedirect.com/science/article/abs/pii/S0749379718316416 29859731 10.1016/j.amepre.2018.03.013 PMC6054889

[6] Fowler JR , Maani EV , Dunton CJ , Gasalberti DP , Jack BW . Cervical cancer. StatPearls. StatPearls Publishing; 2023. Accessed March 1, 2024. https://www.ncbi.nlm.nih.gov/books/NBK431093/ 28613745

[7] Wu CC , Lee MH . Transcatheter arterial embolotherapy: a therapeutic alternative in obstetrics and gynecologic emergencies. Semin Intervent Radiol. 2006;23(3):240‐248. doi:10.1055/s-2006-948761 21326770 PMC3036382

[8] Bohîlțea RE , Dorobaț B , Doldur MM , et al. Uterine arteries embolization‐a rescue tool for acute vaginal bleeding in late stages of gynecologic malignancies. IMR Press. 2022;15:142. doi:10.31083/j.ceog4906142

[9] Gorgens S . The bleeding cervical cancer patient: Systematic Approach and Management. emDOCs.net—Emergency Medicine Education 2022. Accessed March 1, 2024 https://www.emdocs.net/the‐bleeding‐cervical‐cancer‐patient‐systematic‐approach‐and‐management/

[10] Eleje GU , Eke AC , Igberase GO , Igwegbe AO , Eleje LI . Palliative interventions for controlling vaginal bleeding in advanced cervical cancer. Cochrane Database Syst Rev. 2019;3(3):CD011000. doi:10.1002/14651858.CD011000.pub3 30888060 PMC6423555

[11] Alexander‐Sefre F , Covens A , Sonoda Y , et al. Surgical morbidity associated with radical trachelectomy and radical hysterectomy. Gynecol Oncol. 2005;15:450‐454. 10.1016/j.ygyno.2005.11.007 16343604

[12] Kobara H , Mori H , Rafiq K , et al. Application of endoscopic hemostatic forceps for uterine cervical bleeding. Gastrointest Endosc. 2015;81(1):234‐235. 24890426 10.1016/j.gie.2014.04.023

[13] Chattopadhyay SK , Deb Roy B , Edrees YB . Surgical control of obstetrics hemorrhage. Int J Gynecol Obstet. 1990;32:345‐351. 10.1016/0020-7292(90)90112-x 1977629

[14] Evans S , McShane P . The efficacy of internal iliac artery ligation in obstetrics hemorrhage. Surg Gynecol Obstet. 1985;160:250‐253. 3871975

[15] Thavarasah AS , Sivalingam N , Almohdzar SA . Internal iliac and ovarian artery ligation in control of pelvic hemorrhage. Aust N Z J Obstet Gynecol. 1989;29:22‐25. 10.1111/j.1479-828x.1989.tb02870.x 2562595

[16] Yamashita Y , Harada M , Yamamoto H , et al. Transcatheter arterial embolization of obstetrics and gynecological bleeding: efficacy and clinical outcome. Br J Radiol. 1994;67:530‐534. 8032805 10.1259/0007-1285-67-798-530

[17] Pisco JM , Martins JM , Correia MG . Internal iliac artery: embolization to control hemorrhage from pelvic neoplasms. Radiology. 1989;172(2):337‐339. doi:10.1148/radiology.172.2.2748811 2748811

[18] Mihmanli I , Cantasdemir M , Kantarci F , Halit Yilmaz M , Numan F , Mihmanli V . Percutaneous embolization in the management of intractable vaginal bleeding. Arch Gynecol Obstet. 2001;264(4):211‐214. doi:10.1007/s004040000119 11205712

[19] Yalvac S , Kayikcioglu F , Boran N , et al. Embolization of uterine artery in terminal stage cervical cancers. Cancer Investig. 2002;20(5–6):754‐758. doi:10.1081/cnv-120003543 12197232

[20] Hacking C . Superior hypogastric nerve block: Radiology reference article. Radiopaedia. 2022. Accessed March 1, 2024. https://radiopaedia.org/articles/superior‐hypogastric‐nerve‐block#:~:text=Superior%20hypogastric%20nerve%20block%20is,can%20last%20for%20several%20days

[21] Rajan DK , Beecroft JR , Clark TW , et al. Risk of intrauterine infectious complications after uterine artery embolization. J Vasc Interv Radiol. 2004;15(12):1415‐1421. doi:10.1097/01.RVI.0000141337.52684.C4 15590799

